# Supplementary material for: Association between circulating CTRP9 levels and coronary artery disease: a systematic review and meta-analysis
Source: PeerJ. 2024 Nov 18;12:e18488. doi: 10.7717/peerj.18488 (PMC11580665; doi:10.7717/peerj.18488)
Supplement: Supplemental Information 2 [file peerj-12-18488-s002.docx]

1. The rationale for conducting the meta-analysis.
2. The contribution that the meta-analysis makes to knowledge in light of previously published related reports, including other meta-analyses and systematic reviews.

Coronary artery disease (CAD) is a prevalent condition among elderly individuals in China, and new biomarkers that are more sensitive and accurate for early diagnosis of CAD are needed(Keffer 1996; Lobbes et al. 2010). Recently, reports have indicated that low CTRP9 levels can serve as an independent risk factor for CAD(Li et al. 2013). In addition, conflicting results regarding the association between circulating levels of CTRP9 and CAD have also emerged. Therefore, our team had the idea of investigating the association between circulating levels of CTRP9 and CAD.

This study fills the gap in literature by performing meta-analysis on association between circulating CTRP9 levels and CAD. Detailed subgroup analyses of (1) the main characteristics of articles; (2) complications; (3) relevant clinical biochemical indicators; (4) coronary artery lesion; (5) classification of CAD were carried out to illustrate the high heterogeneity. The outcomes confirm the relationship between CTRP9 levels and CAD, which aligned with existing fundamental research, and may contribute to the formation of new CAD biomarker.

Keffer JH. 1996. Myocardial markers of injury. Evolution and insights. *American journal of clinical pathology* 105:305-320. DOI: 10.1093/ajcp/105.3.305

Li M, Lu L, Chen Q, ZHang R, Zhang Q, and Shen W. 2013. Decreased serum CTRP9 levels are associated with coronary artery disease. *Int J Cardiov Dis* 40:323-325. DOI: 10.3969/j.issn.1673-6583.2013.05.018

Lobbes MB, Kooi ME, Lutgens E, Ruiters A, Lima Passos V, Braat S, Rousch M, Ten Cate H, van Engelshoven J, Daemen M, and Heeneman S. 2010. Leukocyte counts, myeloperoxidase, and pregnancy-associated plasma protein a as biomarkers for cardiovascular disease: towards a multi-biomarker approach. *International journal of vascular medicine* 2010:726207. DOI: 10.1155/2010/726207
